# Lipidized PrRP Analog Exhibits Strong Anti-Obesity and Antidiabetic Properties in Old WKY Rats with Obesity and Glucose Intolerance

**DOI:** 10.3390/nu15020280

**Published:** 2023-01-05

**Authors:** Lucia Mráziková, Silvie Hojná, Petra Vaculová, Štěpán Strnad, Vladimír Vrkoslav, Helena Pelantová, Marek Kuzma, Blanka Železná, Jaroslav Kuneš, Lenka Maletínská

**Affiliations:** 1 Institute of Organic Chemistry and Biochemistry, Czech Academy of Sciences, 160 00 Prague, Czech Republic; 2 Institute of Physiology, Czech Academy of Sciences, 142 20 Prague, Czech Republic; 3 Institute of Microbiology, Czech Academy of Sciences, 142 20 Prague, Czech Republic

**Keywords:** diet-induced obesity, Wistar Kyoto rats, glucose intolerance, prolactin-releasing peptide, liraglutide, lipid metabolism, astrocytosis, lipidomics, metabolomics

## Abstract

Prolactin-releasing peptide (PrRP) is an anorexigenic neuropeptide that has potential for the treatment of obesity and its complications. Recently, we designed a palmitoylated PrRP31 analog (palm^11^-PrRP31) that is more stable than the natural peptide and able to act centrally after peripheral administration. This analog acted as an anti-obesity and glucose-lowering agent, attenuating lipogenesis in rats and mice with high-fat (HF) diet-induced obesity. In Wistar Kyoto (WKY) rats fed a HF diet for 52 weeks, we explored glucose intolerance, but also prediabetes, liver steatosis and insulin resistance-related changes, as well as neuroinflammation in the brain. A potential beneficial effect of 6 weeks of treatment with palm^11^-PrRP31 and liraglutide as comparator was investigated. Liver lipid profiles, as well as urinary and plasma metabolomic profiles, were measured by lipidomics and metabolomics, respectively. Old obese WKY rats showed robust glucose intolerance that was attenuated by palm^11^-PrRP31, but not by liraglutide treatment. On the contrary, liraglutide had a beneficial effect on insulin resistance parameters. Despite obesity and prediabetes, WKY rats did not develop steatosis owing to HF diet feeding, even though liver lipogenesis was enhanced. Plasma triglycerides and cholesterol were not increased by HFD feeding, which points to unincreased lipid transport from the liver. The liver lipid profile was significantly altered by a HF diet that remained unaffected by palm^11^-PrRP31 or liraglutide treatment. The HF-diet-fed WKY rats revealed astrogliosis in the brain cortex and hippocampus, which was attenuated by treatment. In conclusion, this study suggested multiple beneficial anti-obesity-related effects of palm^11^-PrRP31 and liraglutide in both the periphery and brain.

## 1. Introduction

Obesity is becoming a major public health problem worldwide, and its prevalence has increased dramatically over the last few decades [1]. Obesity in humans results from a combination of overeating, lower energy expenditure and physical inactivity, but genetic susceptibility could also play a role [1,2,3]. Obesity is a risk factor for insulin resistance (IR) that could result in type 2 diabetes mellitus (T2DM), associated with abnormal insulin secretion and finally chronic hyperglycemia [4,5,6,7]. Moreover, a close relationship between IR and nonalcoholic fatty liver disease (NAFLD) was demonstrated, with a fivefold higher prevalence of NAFLD in patients with T2DM than in patients without T2DM [8].

Anorexigenic peptides are promising tools for the treatment of obesity and related insulin resistance because of their minimal side effects during long-term treatment [9,10,11]. They are produced in the gastrointestinal tract or in the brain and act centrally. They could be modified by lipidization that stabilizes these peptides and enables their central effect after peripheral administration. Liraglutide, a long-acting glucagon-like peptide 1 (GLP-1) receptor agonist modified with palmitic acid, has been successfully used for T2DM (Victoza) and obesity treatment (Saxenda). Another long-acting GLP-1 receptor agonist is semaglutide (Ozempic) with once-weekly administration, which is more convenient for patients [12,13,14,15]. Studies with liraglutide have suggested that this GLP-1 agonist is implicated in maintaining glucose homeostasis by potentiating insulin secretion, inhibiting glucagon output and decreasing appetite [16]. These changes, including a reduction in plasma TG levels, occur independently of diet, whereas the expression of key regulators involved in lipid metabolism probably changes in a diet-dependent manner [17].

Prolactin-releasing peptide (PrRP) is an anorexigenic neuropeptide that has potential for the treatment of obesity and its complications. Recently, we designed PrRP31 analogs palmitoylated at the N-terminus (palm-PrRP31) or position 11 (palm^11^-PrRP31) that are more stable than the natural peptide and able to act centrally after peripheral administration [18,19,20]. Palmitoylated PrRP31 analogs act as antiobesity agents, attenuating lipogenesis in rats [21,22] and mice with high-fat (HF) diet-induced obesity (DIO) [19]. Two weeks of treatment with palm^11^-PrRP31, followed by two weeks of washout in DIO mice decreased BW and improved leptin-related hypothalamic signaling without the yo-yo effect [23].

The aim of this study was to broaden the knowledge from a previous study with WKY rats fed a HF diet for 25 weeks, whose plasma glucose and insulin did not differ from controls fed a standard diet, but who developed a strong intolerance to glucose that was reversed by palm^11^-PrRP31 treatment [21]. In this study, with WKY rats fed a HF diet for 52 weeks, we expected not only intolerance to glucose, but also prediabetes, steatosis in the periphery and insulin resistance-related changes, as well as neuroinflammation in the brain. A potential beneficial effect of 6 weeks of treatment with palm^11^-PrRP31 and liraglutide as comparator was expected.

## 2. Materials and Methods

### 2.1. Substances

An analog of human PrRP palmitoylated at position 11 (palm^11^-PrRP31) with the sequence SRTHRHSMEIK(N-γ-E(*N*-palmitoyl)) TPDINPAWYASRGIRPVGRF-NH_2_ was synthesized at the Institute of Organic Chemistry and Biochemistry, Czech Academy of Sciences (IOCB CAS), Prague, Czech Republic, in the peptide synthesis laboratory by Miroslava Blechová, as previously described [20]. Palm^11^-PrRP31 was dissolved in saline and administered intraperitoneally (IP) at a dose of 5 mg/kg. IP administration of liraglutide (Novo Nordisk, obtained from pharmacy) diluted in saline was used in WKY rats at a dose of 0.2 mg/kg. The dose of palm^11^-PrRP31 used in this study was chosen according to our previous studies [22,24], based on an effective dose of peptides in acute food intake studies.

### 2.2. Experimental Animals

WKY rats were obtained from Charles River (Wilmington, MA, USA). WKY rats were housed under controlled conditions at a constant temperature of 22 ± 2 °C and a fixed 12:12 light:dark cycle. The animals were provided free access to water and a standard rodent chow diet Ssniff^®^ R/M-H (Ssniff Spezialdiäten GmbH, Soest, Germany) containing 33% protein, 9% fat and 58% carbohydrates or an HF diet containing 60% fat, 20% protein and 20% carbohydrates (D12492, Research Diets Inc., New Brunswick, NJ, USA). All animal experiments were performed following the ethical guidelines for work with animals by the Act of the Czech Republic Nr. 246/1992 and were approved by the Committee for Experiments with Laboratory Animals of the CAS.

**Figure 1:**
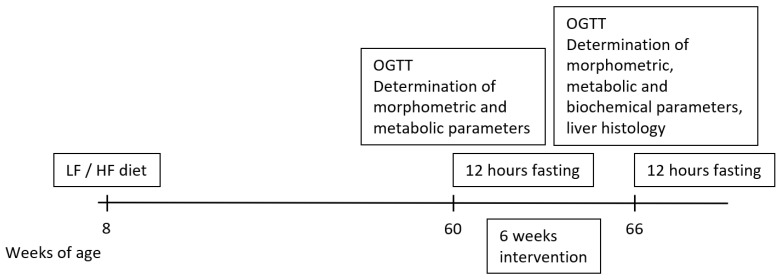
Design of experiment of WKY rats fed HF diet.

A total of 8 WKY rats were fed the Ssniff^®^ diet (WKY LF), while 40 rats were fed an HF diet (WKY HF) for 52 weeks from 8 weeks of age. The design of the experiment is shown in Figure 1. Before the start of treatment, BW was monitored once a week. An oral glucose tolerance test (OGTT) was performed, and plasma samples were collected from tail vessels at 60 weeks of age for determination of the basic biochemical parameters. At the age of 60 weeks, 24 WKY rats on an HF diet with the highest BW were selected and divided into 3 experimental groups. The rats were treated once daily, from Monday to Friday, with saline (WKY HF saline, n = 8), palm^11^-PrRP31 at a dose of 5 mg/kg (WKY HF palm^11^-PrRP31, n = 8) or liraglutide at a dose of 0.2 mg/kg (WKY HF liraglutide, n = 8). Rats fed the Ssniff^®^ diet formed the control group (WKY LF saline, n = 8). During the six-week dosing period, the food intake (FI) and BW were measured twice per week. At the end of the experiment, the rats were fasted overnight, plasma samples were collected from the tail veins for determination of the biochemical parameters, and the OGTT was performed. Then, the animals were deeply anesthetized with pentobarbital (170 mg/kg of BW, Sigma—Aldrich) and transcardially perfused with ice-cold 0.01 mol/L pH 7.4 phosphate buffered saline (PBS) supplemented with heparin (10 U/mL, Zentiva, Prague, Czech Republic). Tissue samples, epididymal white adipose tissue (eWAT), inguinal white adipose tissue (iWAT), liver and half of the brain of all rats were dissected and stored at −80 °C until use. The samples of eWAT and iWAT were homogenized in a Bullet Blender (Next Advance Inc., Averill Park, NY, USA) using lysis buffer [25] and stored at −20 °C until use. The caudate lobes of each liver were used for liver histology. The second brain hemispheres were fixed in paraformaldehyde and used for immunohistochemistry (IHC).

### 2.3. Oral Glucose Tolerance Test

OGTT was performed after overnight fasting. At time point 0, blood was drawn from the tail vein, and a glucose solution at a dose of 2 g/kg of BW was loaded perorally by gavage. The blood glucose concentration was determined in whole blood at 0, 15, 30, 60, 120 and 180 min using a glucometer (Arkray, Tokyo, Japan), and the delta of the area under the curve (AUC) was calculated.

### 2.4. Determination of Biochemical Parameters

Colorimetric assays were used to determine plasma levels of cholesterol (CHOL), triglycerides (TG) (Erba Lachema, Brno, Czech Republic) and free fatty acids (FFAs) (Roche, Mannheim, Germany). The plasma insulin concentration was determined using a radioimmunoassay (RIA) kit (Millipore, St. Charles, MI, USA). Leptin and fibroblast growth factor 21 (FGF21) were determined using mouse and rat enzyme-linked immunosorbent assay (ELISA) kits (Millipore, St. Charles, MI, USA). An ELISA immunoassay kit was used to determine plasma levels of glucagon (Merck, Darmstadt, Germany). Glycated hemoglobin (HbA1c) was determined using the Tina-quant HbA1c Gen. 3 kit (Roche, Mannheim, Germany). All measurements were performed according to the manufacturer’s instructions. The concentration of c-reactive protein (CRP) in fasting plasma was determined using a Mouse CRP ELISA kit (Thermo Scientific, Frederick, MD, USA).

### 2.5. Western Blotting

Samples from eWAT were processed, and western blotting (WB) was performed as previously described [25]. The following primary antibodies were obtained from Cell Signaling Technology (Beverly, MA, USA), and a 1:1000 dilution in 5% milk TBS/Tween-20 was used:, glucose transporter 4 (GLUT4) and substrate of Akt Ser/Thr kinase (AS160).

GAPDH was also obtained from Cell Signaling Technology, Beverly, MA, USA, and a 1:1000 dilution in 5% milk TBS/Tween-20 was used. The following secondary antibodies were used: anti-mouse or anti-rabbit IgG HRP-linked antibody (both from Cell Signaling Technology, Beverly, MA, USA).

### 2.6. Liver Histology

The caudate lobes of each liver were fixed in 4% paraformaldehyde (PFA) in 0.1% mol/L phosphate buffer at pH 7.4. After 24 h in PFA, the liver samples were stored in 70% ethanol at 4 °C until the tissue was processed on a Leica ASP200S tissue processor (Leica Biosystems Inc., Buffalo Grove, IL, USA). Liver samples were wax-penetrated to create paraffin blocks using the paraffin embedding station Leica EG1150H (Leica Biosystems Inc.) and then cut on a Leica RM2255 microtome (Leica Biosystems Inc.). Five-micrometer-thick slices of liver were deparaffinized in xylene and rehydrated in an ethanol range. Slices were stained in hematoxylin and eosin, as described in previous papers [26,27].

### 2.7. NMR-Based Metabolomics in Urine and Plasma

Urine and plasma samples were prepared and measured using a similar procedure as in a previous article [28]. An aliquot of 540 μL urine was mixed with 60 μL 1.5 M phosphate buffer. An aliquot of 300 μL serum was extracted by cold methanol; evaporated supernatant was dissolved in 450 μL D2O with 50 μL phosphate buffer. NMR data were acquired on a 600 MHz Bruker Avance III spectrometer (Bruker BioSpin, Rheinstetten, Germany) equipped with a 5 mm TCI cryogenic probe head. To suppress broad signals of urinary proteins and plasma lipids, NMR analysis was based on a Carr—Purcell—Meiboom—Gill (CPMG) experiment with water presaturation, acquired with the following parameters: presaturation during relaxation delay = 4 s; number of scans = 96 for urine and 256 for plasma samples; number of data points = 64 k; spectral width = 20 ppm; echo time = 0.3 ms; and loop for T2 filter = 126. A short *J*-resolved experiment with presaturation was executed for all samples, and HSQC and TOCSY experiments were performed for selected samples to verify metabolite identification. The spectra, processed in Topspin 3.5 software, were automatically phased, baseline corrected and referenced to the signal of TSP (0.00 ppm); regions with resonances of water, urea (in urine samples) and ethylenediaminetetraacetic acid (in plasma extracts) were excluded before further analysis. Probabilistic quotient normalization (PQN) [29] was performed using the WKY HF saline group as the reference.

Untargeted multivariate analysis, based on the analysis of equidistantly binned (bin width = 0.01 ppm) and Pareto-scaled spectra, was performed in Metaboanalyst 4.0 software [30]. Individual metabolites for the targeted approach were identified using Chenomx software (Chenomx Inc., Edmonton, AB, Canada) and by comparison with the HMDB database (www.hmdb.ca, accessed on 9 September 2021) or previously published data. Based on the Lilliefors test for normality, the significance of changes induced by the HF diet was calculated using an unpaired t test, while the treatment effect was evaluated by one-way ANOVA.

### 2.8. MALDI MSI of Lipids in Liver

Preparation of liver samples for mass spectrometry imaging (MSI) analysis and 2,5-dihydroxybenzoic acid (DHB) sublimation was performed according to our previously published method [31]. For triacylglyceride analysis, the NaDHB solution (10 mg mL^−1^ in acetone) was sprayed using an iMatrixSpray automatic sprayer (Tardo Gmbh, Subingen, Switzerland) with typical spray parameters for dry spraying [32]. Experiments were performed using an ultrafleXtreme matrix-assisted laser desorption/ionization (MALDI) TOF/TOF instrument equipped with a 1 kHz laser (Bruker, Germany) in positive mode in the mass range of 400–2000 Da operating in reflector mode. SCiLS Lab 2016b software (SCiLS GmbH, Bremen, Germany) was used for statistical analysis, as described previously [33].

### 2.9. LC—MS Lipidomics in Liver

Lipids from liver tissue (wet weight 50 mg, n = 5) were extracted using the BUME extraction method [34]. Untargeted lipidomics profiling was performed by coupling an UltiMate 3000 ultrahigh-performance liquid chromatography system to an Orbitrap Fusion Lumos Tribrid mass spectrometer (ThermoFisher Scientific, Waltham, MA, USA) with heated electrospray ionization. Mobile phase A was 60:40 (*v*:*v*) acetonitrile/water, and mobile phase B was 90:10 (*v*:*v*) IPA/acetonitrile. Both contained 10 mM ammonium formate and 0.1% formic acid. The column was a Waters Acquity UPLC BEH C18 (2.1 mm × 50 mm, 1.7 μm) operated at 45 °C and a flow rate of 180 μL/min. The injection volume was 5 μL for positive mode and 10 μL for negative mode. Full scan and MS/MS data acquisitions were obtained in data-dependent analysis mode for both ionization modes separately in the range of 250–1200 Da. An MS resolution of 120,000 and an MS/MS resolution of 15,000 were applied. The electrospray ionization voltage was set at 3.5 kV, and the transfer capillary was set to 320 °C. Lipid identification was performed using LipidSearch 4.2 (ThermoFisher Scientific, Waltham, MA, USA) based on precursor and product ions. Lipid intensities were normalized by internal standard normalization using SPLASH™ Lipidomix^®^ standard (Avanti Polar Lipids, Birmingham, UK). Statistical analysis was performed using MetaboAnalyst software (http://www.metaboanalyst.ca/, accessed on 17 January 2022). Before statistical analysis, the data were subjected to log transformation and Pareto scaling. A volcano plot analysis was used to identify altered lipids between models (fold change > 2 and *p* value < 0.05 by Student’s *t* test).

### 2.10. Determination of mRNA Expression

The liver samples for mRNA determination were processed as previously described [19]. The genes of interest are shown in Table 1. The expression of beta-2-microglobulin (*B2m*) or glucuronidase beta (*GUSB*) was used to compensate for variations in input RNA amounts and the efficiency of reverse transcription.

### 2.11. Immunohistochemistry

PFA-fixed brain hemispheres were processed as previously described [23] with minor modifications. The 50 µm coronal sections were incubated in anti-glial fibrillary acidic protein (GFAP) (Thermo Fisher Scientific, Waltham, MA, USA, 1:200), anti-ionized calcium-binding adaptor molecule 1 (Iba1) (Wako, Osaka, Japan, 1:2000) and anti-doublecortin (DCX) (Cell Signaling Technology 1:600) primary rabbit antibodies overnight at 4 °C. A biotinylated goat anti-rabbit secondary antibody (Vectastain ABC Kit, Vector Laboratories, Burlingame, CA, USA) was used for the incubation of free-floating sections at room temperature for 90 min to perform chromogenic IHC.

### 2.12. Statistical Analysis

The data are presented as the means ± S.E.M.s. Statistical analysis was performed using an unpaired t test or one-way ANOVA, followed by Dunnett’s multiple comparisons test or two-way ANOVA, followed by Bonferroni´s multiple comparisons test, as indicated in the figure legends and tables, with GraphPad Prism software (GraphPad Software, San Diego, CA, USA). The differences were considered significant at *p* < 0.05.

The rate of insulin resistance was expressed with a homeostatic model assessment (HOMA) index calculated as (fasting glucose level, mmol/L) × (fasting insulin level, pmol/L) divided by 22.5 [35].

## 3. Results

### 3.1. Metabolic Parameters of 60-Week-Old WKY Rats Fed a HF or LF Diet

The consumption of the HF diet for 52 weeks resulted in significantly higher BW and plasma leptin in WKY HF rats than in WKY LF rats, while no significant difference in plasma TG and CHOL between WKY HF rats and WKY LF rats was registered (Table 2).

**Figure 2:**
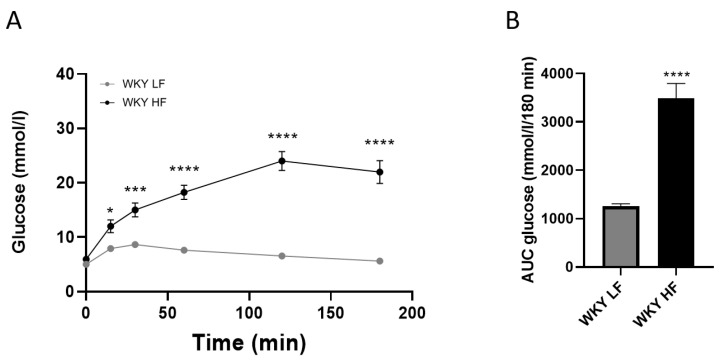
OGTT (**A**) and corresponding AUC (**B**) measured in WKY on LF diet and on HF diet before the treatment (60 weeks of age). Data are presented as means ± S.E.M. Statistical analysis was performed by t-test or two-way ANOVA with Bonferroni’s post hoc test. AUC area under the curve. Significance is * *p* < 0.05, *** *p* < 0.001, **** *p* < 0.0001 vs. WKY LF (n = 8).

WKY HF had significantly increased fasting plasma glucose and insulin, which pointed to insulin resistance that was confirmed by the HOMA index (Table 2). WKY HF showed significant glucose intolerance in the OGTT course (Figure 2) and the corresponding AUC (Figure 2).

### 3.2. Palm^11^-PrRP31 and Liraglutide Treatment Significantly Decreased Body Weight, Food Intake and Plasma Leptin Levels

**Figure 3:**
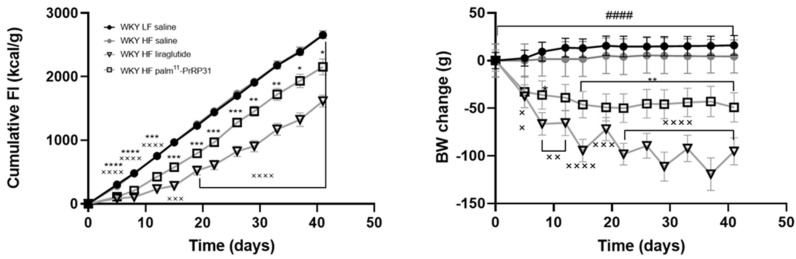
Chronic effect of palm^11^-PrRP31 and liraglutide on cumulative FI and BW change in DIO WKY rats during the treatment (60–66 weeks). Data are presented as means ± S.E.M. Statistical analysis was performed by two-way ANOVA with Bonferroni´s post hoc test. BW body weight, FI food intake. Significance is, ^####^ *p* < 0.0001 WKY HF saline vs. WKY LF saline; ^×^ *p* < 0.05, ^××^ *p* < 0.01, ^×××^ *p* < 0.001, ^××××^ *p* < 0.0001 WKY liraglutide vs. WKY HF saline; * *p* < 0.05, ** *p* < 0.01, *** *p* < 0.001, **** *p* < 0.0001 WKY palm^11^-PrRP31 vs. WKY HF saline (n = 6–8).

During six weeks of treatment, the FI and BW changes of WKY HF saline rats were similar to those of WKY LF saline rats, while treatment with both palm^11^-PrRP31 and liraglutide significantly lowered FI and decreased BW changes (Figure 3).

At the end of the experiment, BW and plasma leptin were significantly higher in WKY HF saline rats than in WKY LF saline rats, but no significant difference in liver weight, plasma TG or CHOL was registered (Table 3). On the contrary, BW and plasma leptin significantly decreased after treatment with both palm^11^-PrRP31 and liraglutide (Table 3). No peptide treatment affected liver weight or plasma CHOL, and only liraglutide treatment decreased plasma TG (Table 3).

### 3.3. Palm^11^-PrRP31 and Liraglutide Treatment Significantly Improved Tolerance to Glucose

**Figure 4:**
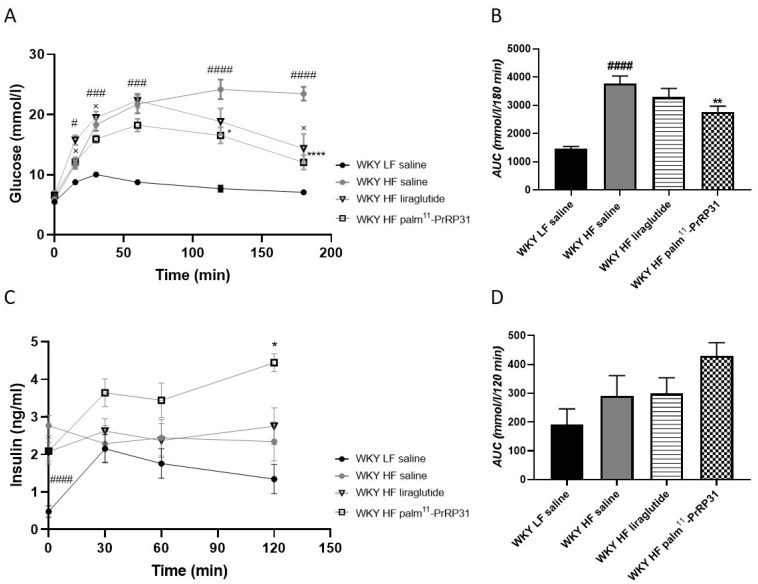
Chronic effect of palm^11^-PrRP31 and liraglutide on OGTT (**A**), corresponding AUC (**B**), plasma levels of insulin during OGTT (**C**) and corresponding AUC (**D**) in DIO WKY rats at the end of the experiment (66 weeks). Data are presented as means ± S.E.M. Statistical analysis was performed by one-way ANOVA with Dunnett´s post hoc test or two-way ANOVA with Bonferroni´s post hoc test. AUC area under the curve. Significance is ^#^ *p* < 0.05, ^###^ *p* < 0.001, ^####^ *p* < 0.0001 WKY HF saline vs. WKY LF saline; ^×^ *p* < 0.05 WKY liraglutide vs. WKY HF saline; * *p* < 0.05, ** *p* < 0.01 **** *p* < 0.0001 WKY palm^11^-PrRP31 vs. WKY HF saline (n = 6–8).

Similar to 60-week-old WKY HF rats before the treatment, 66-week-old WKY HF saline rats again showed strong glucose intolerance in the OGTT, compared to WKY LF saline rats at the end of the experiment (Figure 4). Palm^11^-PrRP31 treatment very significantly lowered the glucose area under the curve in the OGTT (Figure 4B). The insulin area under the curve did not differ among the experimental groups (Figure 4D).

Even though no significant difference in fasted plasma glucose between the WKY LF saline and WKY HF saline groups was obvious at the end of the experiment, HbA1c, plasma insulin, HOMA index, plasma glucagon and FGF21 were higher in WKY HF saline rats than in WKY LF saline rats (Table 3). Treatment with both palm^11^-PrRP31 and liraglutide had no effect on fasted plasma glucose or insulin. Liraglutide treatment significantly decreased HbA1c, as well as plasma glucagon and FGF21 (Table 3). No treatment affected the HOMA index (Table 3).

**Figure 5:**
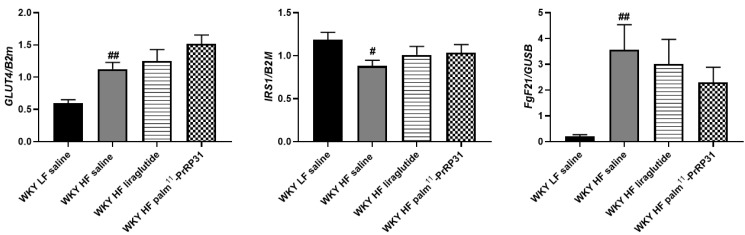
Chronic effect of palm^11^-PrRP31 and liraglutide on genes involved in glucose metabolism measured by mRNA expression in eWAT (*GLUT4*, *IRS1*) or in the liver (*FGF21*) at the end of the experiment (66 weeks). Data are presented as means ± S.E.M. Statistical analysis was performed by one-way ANOVA with Dunnett´s post hoc test. GLUT4 glucose transporter 4, IRS1 insulin receptor substrate 1, FGF21 fibroblast growth factor 21. Significance is ^#^ *p* < 0.05, ^##^ *p* < 0.01 WKY HF saline vs. WKY LF saline (n = 5–6).

**Figure 6:**
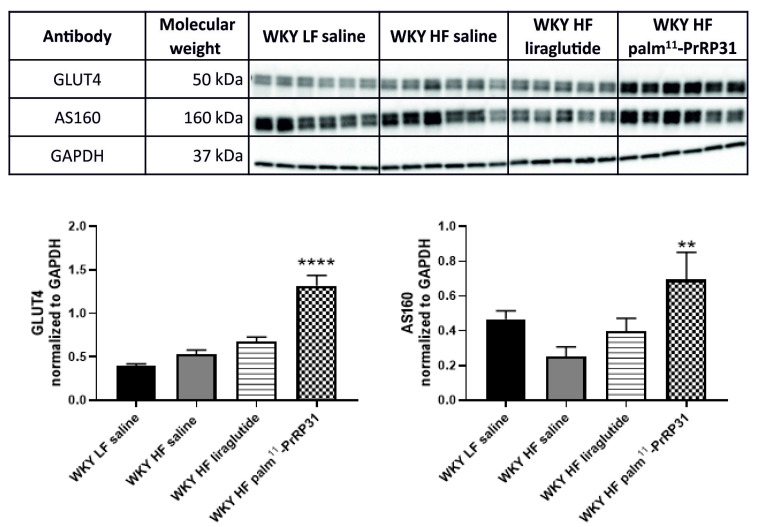
Chronic effect of palm^11^-PrRP31 and liraglutide on glucose metabolism measured by western blot in eWAT at the end of the experiment (66 weeks). Data are presented as means ± S.E.M. Statistical analysis was performed by one-way ANOVA with Dunnett´s post hoc test. IRS1 insulin receptor substrate 1, AS160 Akt substrate of 160 kDa, GLUT4 Glucose transporter 4. Significance is ** *p* < 0.01, **** *p* < 0.0001 WKY palm^11^-PrRP31 vs. WKY HF saline (n = 5–6).

In eWAT, *Irs-1* mRNA expression was lower in WKY HF saline compared to WKY LF saline (Figure 5). Even though GLUT4 mRNA expression in eWAT was higher in WKY HF saline than in WKY LF saline, GLUT4 protein did not differ. Palm^11^-PrRP31 treatment increased eWAT GLUT4 protein and its accompanying protein AS160 (Figure 5 and Figure 6).

Liver *FGF21* mRNA expression was significantly enhanced in WKY HF saline compared to WKY LF saline and stayed unaffected by both peptide treatments (Figure 5), but plasma FGF21 was attenuated by liraglutide treatment (Table 3).

### 3.4. NMR-Based Metabolomics

#### 3.4.1. The Impact of a HF Diet on Urine and Plasma Metabolic Profiles

The impact of the HF diet on the metabolic composition of urine was evaluated using the samples collected before the therapy. The PCA model built from the binned spectra did not detect any outliers. The PLS-DA score plot showed clear separation of the lean and obese groups (see Supplementary Figure S1), based on the increase in taurine, glucose and tartrate and the decrease in creatinine, hippurate, citrate, phenylacetylglycine, 2-oxoglutarate and isovalerylglycine. Subsequently, univariate analysis was applied to evaluate the changes in 49 metabolites quantified in urine samples. A 2-tailed unpaired t test revealed significant changes in 33 metabolites (see Supplementary Table S1). Plasma characterization of the HF diet-fed group was based on samples collected during dissections at the end of the experiment. Neither the PCA nor the PLS-DA model showed satisfactory LF and HF diet-fed group separation. This finding indicates that no combination of plasma metabolites is sufficient to clearly distinguish obese and lean animals. Nevertheless, the results of univariate analysis by t test showed significant differences between the WKY LF and HF groups in 10 metabolites (see Supplementary Figure S1 and Table S1).

#### 3.4.2. The Impact of Palm^11^-PrRP31 and Liraglutide Treatment on Urine and Plasma Metabolic Profiles

None of the therapies induced changes intense enough to differentiate between treated and untreated (HF saline) groups in PLS-DA models. Subsequently, changes between the palm^11^-PrRP31, liraglutide and saline-treated HF groups were evaluated in individual metabolites by parametric ANOVA. Even though the urine profiles of obese and lean rats significantly differed, the treatment affected only a few metabolites, as shown in Table 4. Plasma profiles seem to be more sensitive to the treatment with the bigger impact of the palm^11^-PrRP31 therapy. All significant treatment-induced changes are shown in Table 4.

### 3.5. No Liver Steatosis but Different Lipid Profiles in 66-Week-Old WKY Rats Fed a HF Diet

**Figure 7:**
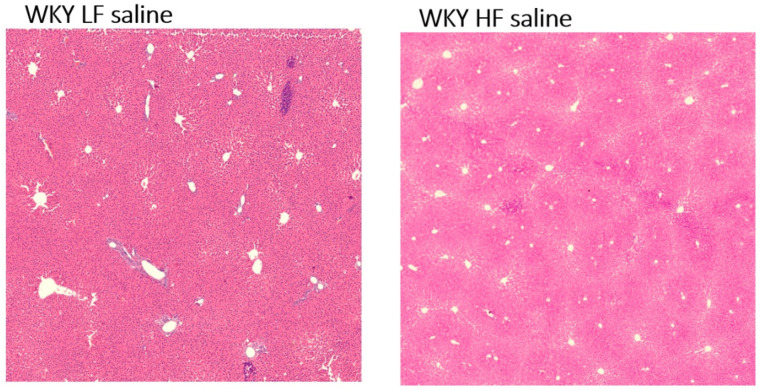
Liver histology, representative photomicrographs of WKY LF saline and WKY HF saline (n = 6). Eosin-hematoxylin staining. The magnification of photomicrographs is 200×.

As shown in Table 3, WKY HF saline rats and WKY LF saline rats had surprisingly similar liver weights. No treatment affected liver weight. Regarding liver histology, no accumulation of fat droplets or liver steatosis was detected in either WKY LF saline or WKY HF saline rats (Figure 7).

**Figure 8:**
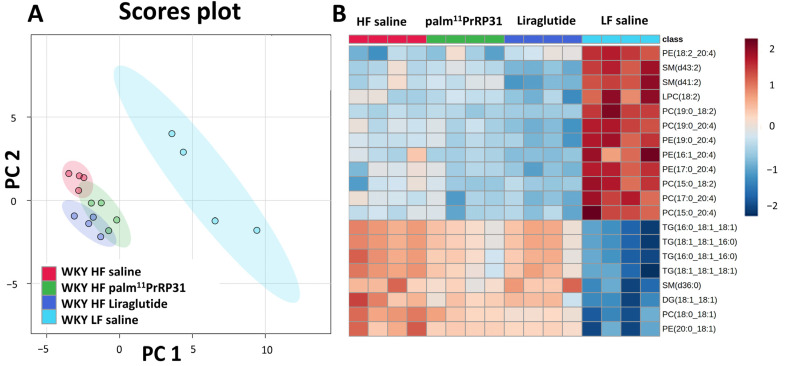
(**A**) PCA score plot based on lipid profiling of WKY LF saline, WKY HF saline, WKY HF liraglutide and WKY HF palm^11^-PrRP31 rats. (**B**) Heatmap of top 20 lipid features based on one-way ANOVA rankings.

Using untargeted lipidomic profiling of liver homogenates, we identified a total of 145 lipids. PCA (Figure 8A) based on lipid profiling revealed a clear separation between WKY HF saline and WKY LF saline. The heatmap in Figure 8B shows the comparison of intensities of the top 20 lipid features, based on statistical significance according to one-way ANOVA. Both methods clearly show changes in liver lipid profiles between WKY HF saline and WKY LF saline rats. In the volcano plot (Supplementary Figure S2), the highest change caused by HF diet feeding was the upregulation of triacylglycerols, diacylglycerols (DG) and phosphatidylcholines (PC) containing mostly saturated—SFA (16:0, 18:0) and monounsaturated fatty acids—MUFA (16:1, 18:1). On the contrary, HF diet feeding downregulated mostly low-abundance phospholipids containing polyunsaturated fatty acids (PUFAs) (18:2, 20:4) and, surprisingly, frequently fatty acids with odd numbers of carbon atoms.

Treatment with palm^11^-PrRP31 and liraglutide significantly downregulated some TG, which suggested a positive effect on the liver lipidome (volcano plot, Supplementary Figures S3 and S4). However, PCA showed that palm^11^-PrRP31-, liraglutide- and saline-treated models on HF diets were separated, but clustered next to each other, indicating overall lipid profile similarity.

**Figure 9:**
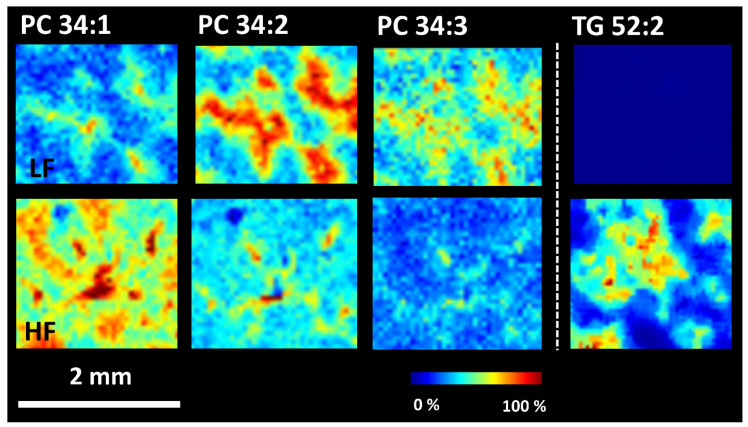
MALDI MSI analysis of WKY rats on LF and HF diet. Ion images of phophatidylcholines (PC 34:2, *m*/*z* 796.5; PC 34:1, *m*/*z* 798.5, [M + K]^+^) and triacylglycerol (TG 52:2, *m*/*z* 897.7, [M + Na]^+^) were obtained in positive ion mode at spatial resolution 40 μm.

MALDI MSI analysis revealed changes in lipid distribution in the liver resulting from HF diet feeding. ROC analysis was used to identify changes in the m/z values. The most significant differences were found in the upregulation of phosphatidylcholines (PC 34:1, 36:1) and triacylglycerols (TG 50:2, 52:3, 52:2, 54:2) in WKY HF saline, compared to WKY LF saline. Examples of the lipid distributions in WKY rats fed the HF and LF diets are shown in Figure 9. The results correspond to untargeted lipidomic profiling. Consistent with data from lipidomic profiling, the increased abundance of monounsaturated lipid PC 34:1 and decreased abundance of more unsaturated PC 34:2 and PC 34:3 in the liver of WKY HF saline compared to WKY LF saline were observed. The accumulation of TG 52:2 in the livers of WKY rats fed a HF diet also supported the lipidomics results.

### 3.6. Liver Lipogenesis Was Stimulated in 66-Week-Old WKY Rats Fed a HF Diet, and Palm^11^-PrRP31 Treatment Attenuated the mRNA Expression of Genes Regulating Liver Lipogenesis

**Figure 10:**
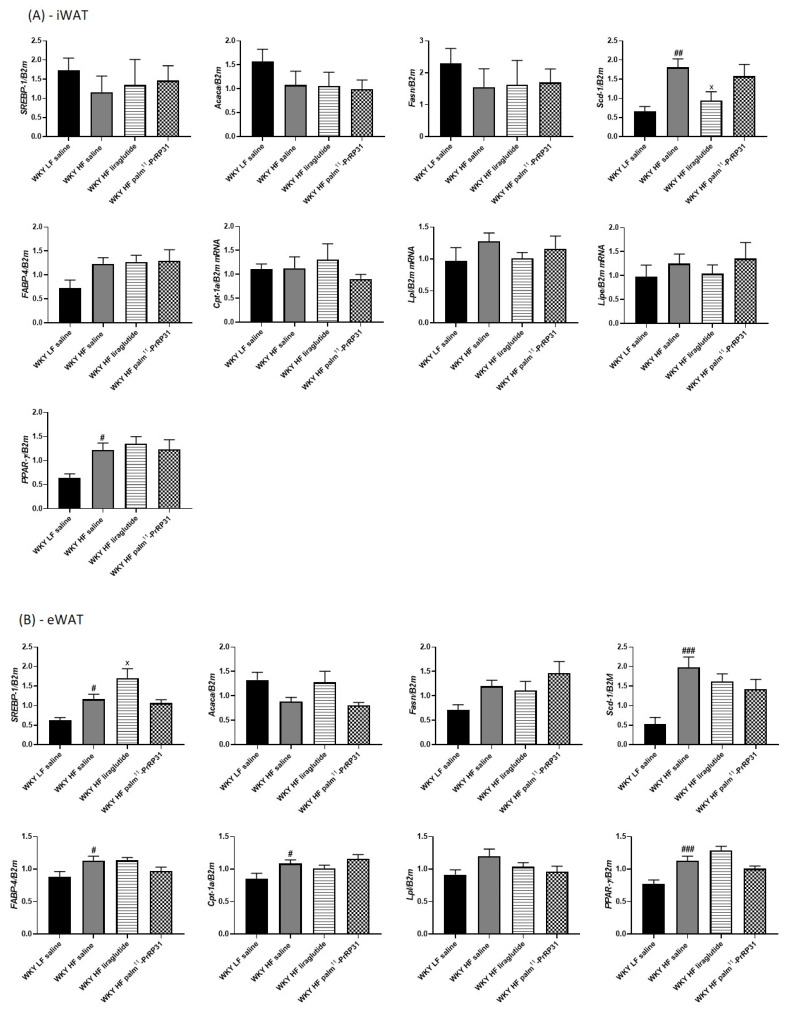

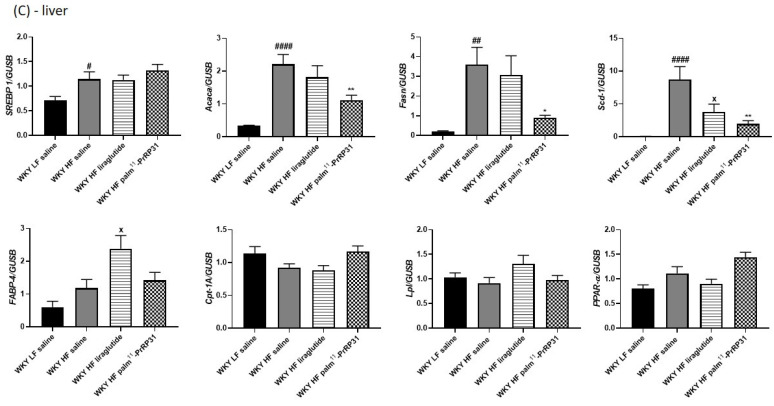
Chronic effect of palm^11^-PrRP31 and liraglutide on mRNA expression in iWAT (**A**), eWAT (**B**) and liver (**C**) in DIO WKY rats at the end of the experiment (66 weeks). Data are presented as means ± S.E.M. Statistical analysis was performed by one-way ANOVA with Dunnett´s post hoc test. *Acaca* acetyl-CoA carboxylase, eWAT epididymal white adipose tissue, *FABP-4* fatty acid-binding protein 4, *Fasn* fatty acid synthase, *Cpt-1a* carnitine palmitoyltransferase 1a, *Lipe* lipase E, *Lpl* lipoprotein lipase, *Ppar-α*/*γ* peroxisome proliferatore-activated receptor alpha/gamma, iWAT inguinal adipose tissue, *Scd-1* stearoyl-CoA desaturase 1, *SREBP-1* sterol regulatory element binding protein 1. Significance is ^#^ *p* < 0.05, ^##^ *p* < 0.01, ^###^ *p* < 0.001 ^####^ *p* < 0.0001 WKY HF saline vs. WKY LF saline; ^×^ *p* < 0.05, WKY liraglutide vs. WKY HF saline; * *p* < 0.05, ** *p* < 0.01, WKY palm^11^-PrRP31 vs. WKY HF saline (n = 6–8).

HF diet feeding supported liver lipogenesis by enhanced mRNA expression of *Acaca*, *Fasn*, of their transcription factor *SREBP-1* and very significantly of stearoyl desaturase-1 (*Scd-1*) in WKY HF saline, compared to WKY LF saline (Figure 10C). Treatment with palm^11^-PrRP31 significantly attenuated the mRNA expression of *Acaca*, *Fasn* and *Scd-1*, but not *SREBP-1*, while liraglutide treatment decreased *Scd-1* mRNA expression only (Figure 10C). On the contrary, the HF diet also significantly enhanced liver *Fgf21* mRNA expression in WKY HF saline, compared to WKY LF saline (Figure 5), but the mRNA expression of *PPAR-α* and *CPT-1*, which are involved in fatty acid oxidation, did not differ among the experimental groups (Figure 10).

Regarding lipogenesis in adipose tissue, the mRNA expression of *Scd-1* was increased in both iWAT and eWAT, while that of *SREBP-1* was higher only in eWAT of WKY HF saline rats, rather than WKY LF saline rats. Peroxisome proliferator-activated receptor γ (*PPAR-γ*) mRNA expression was higher in WKY HF saline than in WKY LF saline in both eWAT and iWAT. The mRNA expression of *FABP-4* and *Cpt-1a*, genes involved in lipolysis, was increased in eWAT only. Liraglutide treatment decreased the mRNA expression of *Scd-1* in iWAT, while palm^11^-PrRP31 treatment had no effect on lipogenesis-related genes mRNA expression in adipose tissue (Figure 10A,B).

### 3.7. Palm^11^-PrRP31 Attenuated Astrocytosis in the Isocortex and Hippocampus

**Figure 11:**
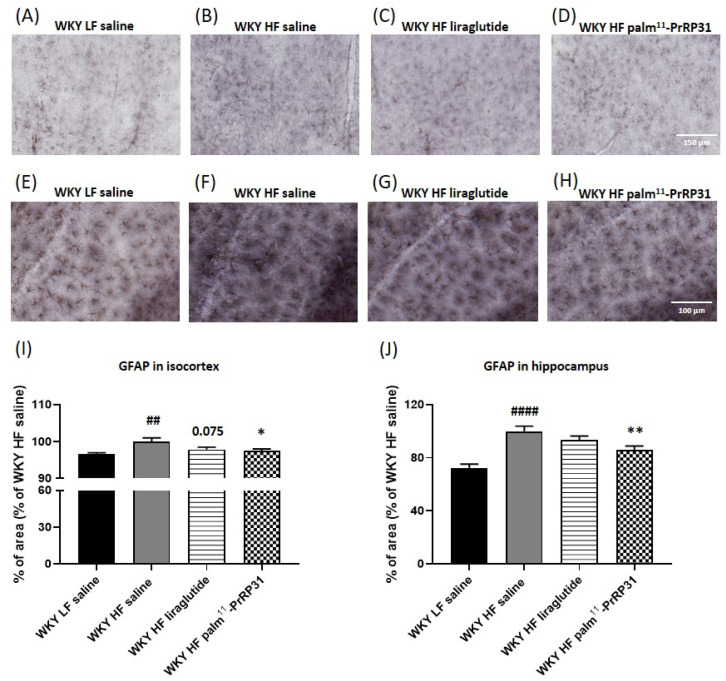
Reduction of astrocytosis in isocortex and hippocampus of WKY HF rats after treatment with palm^11^-PrRP31. (**A**–**D**), representative photomicrographs of the isocortex of the treated and control WKY HF saline rats and their age-matched WKY LF controls immunohistochemically stained for the astrocyte marker GFAP. (**E**–**H**), detailed photomicrographs illustrating the hippocampal astrocytosis. (**I**,**J**), quantification of immunohistochemical staining in the isocortex (**I**) and hippocampus (**J**). The data are presented as means ± S.E.M. Statistical analysis was performed by one-way ANOVA with Dunnett´s post hoc test. Statistical significance is ^##^ *p* < 0.01, ^####^ *p* < 0.0001 WKY HF saline vs. saline-treated WKY LF; * *p* < 0.05, ** *p* < 0.01 WKY palm^11^-PrRP31 vs. WKY HF saline (n = 5–6).

**Figure 12:**
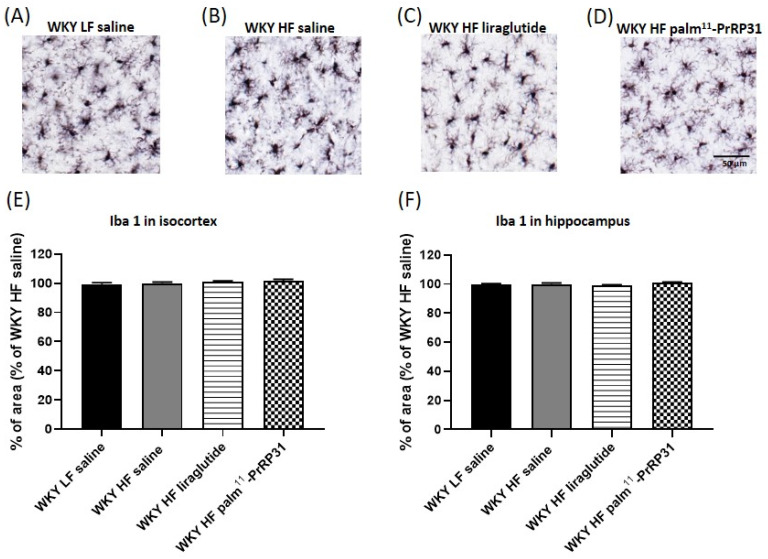
No visible microgliosis in the isocortex and hippocampus of WKY rats after 52 weeks on high-fat diet. Treatment with liraglutide and palm^11^-PrRP31 has no effect on Iba 1 staining. (**A**–**D**), representative photomicrographs of the microglial marker Iba 1 in the isocortex, quantification of Iba 1 immunohistochemical staining in the isocortex (**E**) and hippocampus (**F**). The data are presented as means ± S.E.M. Statistical analysis was performed by one-way ANOVA with Dunnett´s post hoc test (n = 5–6 rats per group).

The photomicrographs of immunohistochemically stained brain sections (Figure 11) show that WKY HF saline had a higher GFAP reactivity than WKY LF saline in both the isocortex and hippocampus. The percentage of the cortical and hippocampal areas stained for the astrocyte marker GFAP was significantly reduced after treatment with palm^11^-PrRP31. Liraglutide treatment tended to reduce astrocytosis in the isocortex (*p* = 0.075), but not in the hippocampus. Immunohistochemical staining for Iba 1, a marker of both resting and activated microglia (Figure 12), shows no visible clusters of activated microglia in any experimental group.

Immunostaining for doublecortin (DCX), a marker of newly generated neurons (DCX^+^), did not show significant changes in the number of doublecortin-positive cells among the experimental groups (Supplementary Figure S5).

## 4. Discussion

This study with WKY rats more than 1 year old fed a HF diet for 52 weeks is a follow-up of our previous study with WKY rats fed a HF diet for 25 weeks that did not develop hyperinsulinemia or hyperglycemia, but a strong intolerance to glucose that was reversed by palm^11^-PrRP31 treatment [21]. In rats of doubled age in this study, we expected not only intolerance to glucose, but also prediabetes and steatosis in the periphery and insulin resistance-related changes in the brain. Under such conditions, we aimed to test the potential beneficial effect of 6 weeks of treatment with the anti-obesity peptide palm^11^-PrRP31, using liraglutide as a comparator.

In our previous studies, the stability of PrRP analogs was determined in mice [19] and rats [36], and significantly increased stability of lipidized PrRP analogs was found.

A decrease in body weight by treatment with anti-obesity substances could beneficially affect sensitivity to insulin and tolerance to glucose, and it might positively affect lipid metabolism in the liver.

Obesity resulting from the HF diet feeding in this study was mirrored by an enhanced leptin level in WKY rats 60 and 66 weeks old, before and at the end of the experiment, respectively. Analogously to our two previous studies with palm-PrRP31 and palm^11^-PrRP31 [21], palm^11^-PrRP31 treatment attenuated body weight and leptin levels in this study. Similarly, our comparator liraglutide reduced food intake and body weight, together with plasma leptin levels in DIO rats, which is consistent with other studies [37,38].

Obesity-induced insulin resistance was proven by increased insulin levels in WKY rats fed a HF diet, both 60 and 66 weeks old, which resulted in a high HOMA index. An augmented plasma FGF21 in WKY HF saline, compared to WKY LF saline, at the end of the experiment suggested insulin state resistance, as well. It is known that FGF21 plasma levels are increased in insulin resistance states, but it is still not clear whether this occurs because of resistance to or compensatory secretion of FGF21 (reviewed by Szczepanska et al. [39]).

Fasted plasma glucose in HF-diet-fed rats was higher than in LF-diet-fed 60-week-old rats, but still did not reach hyperglycemia, and at the end of the experiment, 66-week-old WKY HF saline did not differ from WKY LF saline in fasted glucose, but had an increased blood HbA1c. Unlike palm^11^-PrRP31, liraglutide treatment decreased HbA1c, plasma glucagon and FGF21, which points to its anti-diabetic effect. The OGTT before and at the end of the experiment pointed to a robust intolerance to glucose in HF-diet-fed rats, analogous to 26-week-old WKY rats fed a HF diet [21]. Palm^11^-PrRP31 treatment attenuated intolerance to glucose in this study, as it did previously [21], while liraglutide had no effect. Interestingly, in Spraque Dawley rats, HF diet feeding did not provoke glucose intolerance or an increase in plasma fasted insulin or glucose [22].

Surprisingly, in this study, the liver weight and histology of 66-week-old WKY HF saline did not differ from WKY LF saline. Similar results were reported recently with WKY rats that did not increase their liver weight and lipid content after eight weeks of HF diet feeding [40]. However, albino Wistar rats fed the same diet for eight weeks, as was used in our study, had an enhanced liver lipid content; yet, their liver weight was not reported [41].

Both TG and cholesterol plasma levels did not differ between HF- and LF-diet-fed rats before and at the end of the experiment, which suggests that HF diet feeding did not affect lipid transport from the liver. The only exception was a group treated with liraglutide, which was characterized by a reduced plasma level of triglycerides. This TG-lowering effect was also seen by Decara et al. in both lean and obese Sprague—Dawley rats [17].

The urinary and plasma metabolic profiles of the HF WKY group at 60 weeks are analogous to those of the 25-week-old rats published in our previous study [21]. High-fat diet feeding induced changes mainly in the nicotinamide pathway, TCA cycle, glucose and polyamine metabolism, and excretion of microbial cometabolites, which are consistent with the development of obesity. Palm^11^-PrRP31 and liraglutide treatment significantly reduced the levels of some metabolites, which were initially elevated in the WKY HF saline group as a result of HF diet feeding. This is primarily a decrease in creatinine, taurine and β-alanine concentrations in urine and a decrease in creatine, 3-hydroxybutyrate, saccharides and fumarate in plasma.

Although the results did not demonstrate the development of steatosis in WKY HF rats, some metabolic changes may indicate improvement of impaired liver function after palm^11^-PrRP31 and liraglutide administration. In this study, the increased concentration of β-alanine in the WKY HF group was attenuated by both types of therapy. Increased levels of the β-alanine precursor N-carbamoyl-β-alanine were reported in several experimental models of obesity [42,43,44]. Because β-alanine is also involved in protecting the liver from hypoxic damage in a rat model [45], its increased concentration in obese WKY rats could therefore compensate for liver damage with the progression of obesity. Increased taurine excretion may serve as an indicator of liver damage [46], which can be caused by inflammation related to lipid β-oxidation and oxidative stress [47]. The depletion of urinary taurine after treatment with liraglutide and palm^11^-PrRP31 (insignificant trend) is consistent with the effect of liraglutide [21] and two analogs of palmitoylated PrRP [20] on obese C57BL/6 mice. It can be concluded that the β-alanine and taurine reduction with palm^11^-PrRP31 and liraglutide treatment could reflect an improvement in liver function.

Despite no signs of steatosis, liver lipogenesis was significantly enhanced, according to increased gene transcription of the lipogenesis enzymes *Acaca* and *Fasn*, their transcription factor *Sreb-1* and the desaturase *Scd*-1 in WKY HF saline rats, compared to WKY LF saline rats. Palm^11^-PrRP31 treatment lowered the mRNA expression of the genes involved in lipogenesis, similar to WKY rats [21] or palm-PrRP31 in Sprague Dawley rats [22] or in mice [19], all fed a HF diet. Liraglutide treatment is also associated with decreased expression of genes/proteins involved in lipogenesis in the liver or kidney, suggesting a recovery of lipid homeostasis. Target genes/proteins differ according to the rat DIO model used experimentally [17].

The main effect of GLP-1 and its lipidized analog liraglutide is augmentation of glu-cose-stimulated insulin secretion. Insulin has mostly an anabolic effect, and in the liver, it supports lipogenesis. Liraglutide induces insulin secretion that increases liver lipogenesis and, in addition, the liraglutide central anorexigenic effect decreases intake of high fat food and resulting lipid deposition in the liver [16]. As palm^11^-PrRP31 does not act as an insulin secretagogue, its effect is only anorexigenic and, thus, more attenuating on liver lipogenesis.

Very significantly enhanced *Scd-1* mRNA expression points to desaturation of saturated fatty acids and their easier oxidation. Unsaturated fatty acids oxidize better and are less likely to accumulate than saturated fatty acids [48]. A beneficial effect of SCD-1 in the liver is supported by the fact that increased hepatocyte apoptosis, lipotoxicity and steatosis occur if SCD-1 is pharmacologically inhibited [49]. In addition, we hypothesized that the paracrine action of FGF21 in the liver, whose liver mRNA expression was enhanced by HF diet feeding, could stimulate hepatic fatty acid oxidation and, thus, utilize excessive fatty acids from lipogenesis, which was also enhanced by HF diet feeding. In rodents, an elevated FGF21 level induced by obesogenic diet feeding has been recently attributed to low protein and/or high sugar content, rather than high fat content in a diet [50,51]. The HF diet used in this study (60% fat as energy content) contained less protein (20% of energy content) than the LF diet (33% of energy content) and sucrose (20% of energy content) as the only saccharide. It seems that such a diet could trigger an FGF21 rise in WKY rats.

Despite the fact that liver weight and liver histology were similar in WKY HF saline and WKY LF saline and steatosis was not detected, a robust difference was found in the liver lipid profile. Phosphatidylcholines, diacylglycerols and triacylglycerols containing saturated and monosaturated fatty acids were upregulated by HF diet feeding, while phospholipids with polyunsaturated fatty acids and saturated fatty acids of odd carbon number were downregulated, as to the lipid profile data. Similar results were reported in a human study [52], where HF diet feeding increased liver TG containing at least one unsaturated fatty acid, while the LF diet enhanced TGs with polyunsaturated fatty acids. Analogously, in a study with WKAM/HkmSlc rats [53], a HF diet increased saturated fatty acids in TGs, but attenuated PUFAs in phosphatidylcholines, phosphatidylinositols and phosphatidylserines, and PUFA content was found to be negatively related to obesity.

Obesity-related low-grade inflammation did not come to light in the periphery, as plasma CRP in WKY HF saline was not higher, compared to WKY LF saline rats (Supplementary Figure S6). However, astrocytosis, but not microgliosis, in the brain cortex and hippocampus was obvious in WKY HF saline rats compared to WKY LF saline rats. Palm^11^-PrRP31 treatment lowered astrocytosis, as it did previously in APP/PS1 mice overexpressing chimeric amyloid precursor protein with mutations found in familial Alzheimer’s disease and presenilin [54].

Acute in vivo experiments demonstrated that lipidized PrRP analogs have central anorexigenic effects after peripheral administration. There was a significant and dose-dependent decrease in food intake in lean overnight-fasted or freely fed mice after subcutaneous (SC) injection of palm-PrRP31 or palm^11^-PrRP31. Moreover, neuronal activity (manifested by increased expression of the immediate early gene c-Fos in brain areas related to food intake regulation) was significantly increased in specific brain nuclei or in areas such as the Arc, PVN, DMN and NTS after SC application of palm-PrRP31 and palm^11^-PrRP31, but not after natural PrRP [55]. Furthermore, double c-Fos-GPR10 immunostaining in the brainstem C1/A1 cell group indicated that neurons containing GPR10 receptors are activated after administration of palmitoylated PrRP [56]. Finally, palmitoylated analogs of PrRP affected lipid metabolism in adipose tissue and the liver by suppressing lipid synthesis and increasing lipid degradation. The effect of palm^11^-PrRP31 seems to be anorexigenic and attenuating liver lipogenesis.

## 5. Conclusions

WKY rats that were 66 weeks old and were fed a HF diet for 52 weeks developed obesity that was significantly decreased by both palm^11^-PrRP31 and liraglutide treatment. In parallel, they showed robust glucose intolerance that was attenuated by palm^11^-PrRP31, but not by liraglutide intervention. On the contrary, liraglutide had a beneficial effect on the insulin resistance parameters HbA1c, plasma glucagon and FGF21. Despite obesity and prediabetes, WKY rats did not develop liver steatosis owing to HF diet feeding, even though liver lipogenesis was enhanced. Plasma TG and cholesterol were not increased by HFD feeding, which points to unincreased lipid transport from the liver. It seems that liver lipid oxidation was probably enhanced, as well, when lipid accumulation in the liver did not occur. A large change altered by HF diet feeding was the liver lipid profile, which remained unaffected by palm^11^-PrRP31 or liraglutide treatment. Changes in urine and plasma metabolic profiles revealed that palm^11^-PrRP31 and liraglutide treatment significantly reduced the levels of some metabolites, which were initially elevated in the WKY HF saline group as a result of HF diet feeding. The HF-diet-fed WKY rats showed astrogliosis in the brain isocortex and hippocampus, which was attenuated by treatment. Overall, this study suggested multiple beneficial anti-obesity-related effects of palm^11^-PrRP31 and liraglutide in both the periphery and brain.

## Figures and Tables

**Table 1:** mRNA expression of the following genes was analyzed in adipose tissues and in the liver.

ADIPOSE TISSUE	*Acaca*	LIVER	*Acaca*
	*Cpt-1a*		*Cpt-1*
	*FABP-4*		*FABP-4*
	*Fasn*		*Fasn*
	*GLUT-4*		*Lpl*
	*Irs-1*		*Ppar-α*
	*Lipe*		*Scd-1*
	*Lpl*		*SREBP-1*
	*Ppar-γ*		
	*Scd-1*		
	*SREBP-1*		

*Acaca* acetyl-CoA carboxylase, *Cpt-1a* carnitine palmitoyltransferase 1a, *FABP-4* fatty acid-binding protein 4, *Fasn* fatty acid synthase, *GLUT-4* glucose transporter type 4, *Irs-1* insulin receptor substrate 1, *Lipe* lipase E, *Lpl* lipoprotein lipase, *Ppar-α*/*γ* peroxisome proliferatore-activated receptor alpha/gamma, *Scd-1* stearoyl-CoA desaturase 1, *SREBP-1* sterol regulatory element binding protein 1.

**Table 2:** Morphometric and metabolic parameters analyzed in fasted plasma of WKY LF and WKY HF before the treatment (60 weeks of age).

Age	60 Weeks	
Group	WKY LF	WKY HF
BW (g)	439.25 ± 6.85	589.42 ± 6.81 ****
Glucose (mmol/L)	5.00 ± 0.25	6.11 ± 0.09 ****
Insulin (ng/mL)	0.53 ± 0.12	2.95 ± 0.19 ***
HOMA index	20.85 ± 5.07	133.46 ± 11.60 ****
Leptin (ng/mL)	2.29 ± 0.28	33.02 ± 1.75 ****
TG (mmol/L)	0.46 ± 0.06	0.47 ± 0.02
CHOL (mmol/L)	3.51 ± 0.25	3.20 ± 0.09

Data are presented as means ± S.E.M. Statistical analysis was performed by *t*-test. BW body weight, TG triglycerides, CHOL cholesterol. Significance is *** *p* < 0.001, **** *p* < 0.0001 vs. WKY LF (n = 8, 24).

**Table 3:** Morphometric and metabolic parameters analyzed in fasted plasma of WKY LF saline, WKY HF saline, WKY HF liraglutide and WKY HF palm^11^-PrRP31 at the end of the experiment (66 weeks of age).

Age	66 Weeks			
Group	WKY LF Saline	WKY HF Saline	WKY HF Liraglutide	WKY HF Palm^11^-PrRP31
BW (g)	444.00 ± 8.55	591.00 ± 12.37 ^####^	456.83 ± 11.77 ^xxxx^	519.14 ± 9.05 ***
Liver weight (g)	10.81 ± 0.55	11.20 ± 0.435	9.76 ± 0.40	10.42 ± 0.34
Leptin (ng/mL)	3.13 ± 0.60	24.52 ± 2.22 ^####^	12.60 ± 2.13 ^xxx^	15.14 ± 2.07 **
TG (mmol/L)	0.54 ± 0.06	0.56 ± 0.03	0.38 ± 0.03	0.59 ± 0.05
CHOL (mmol/L)	2.79 ± 0.12	2.64 ± 0.15	2.47 ± 0.13	2.50 ± 0.15
Glucose (mmol/L)	5.49 ± 0.17	5.98 ± 0.17	6.62 ± 0.65	6.56 ± 0.28
HbA1c (mmol/mol)	2.00 ± 0.09	2.62 ± 0.13 ^##^	1.86 ± 0.13 ^xxx^	2.27 ± 0.08
Insulin (ng/mL)	0.48 ± 0.16	2.77 ± 0.27 ^####^	2.07 ± 0.25	2.09 ± 0.35
HOMA index	20.65 ± 6.69	127.25 ± 13.29 ^####^	104.73 ± 15.00	116.58 ± 19.38
Glucagon (pg/mL)	4.33 ± 0.05	4.58 ± 0.04 ^##^	4.38 ± 0.04 ^x^	4.41 ± 0.07
FGF21 (pg/mL)	262.96 ± 36.90	607.63 ± 147.23 ^#^	173.85 ± 31.75 ^x^	512.64 ± 149.38

Data are presented as means ± S.E.M. Statistical analysis was performed by one-way ANOVA with Dunnett´s post hoc test. BW body weight, TG triglycerides, CHOL cholesterol, HbA1c glycated hemoglobin, FGF21 fibroblast growth factor 21. Significance is ^#^ *p* < 0.05, ^##^ *p* < 0.01 ^####^ *p* < 0.0001 WKY HF saline vs. WKY LF saline; ^×^ *p* < 0.05, ^×××^ *p* < 0.001, ^××××^ *p* < 0.0001 WKY liraglutide vs. WKY HF saline; ** *p* < 0.01, *** *p* < 0.001 WKY palm^11^-PrRP31 vs. WKY HF saline (n = 6–8).

**Table 4:** Urinary and serum metabolites significantly changed in WKY HF palm^11^-PrRP31, WKY HF liraglutide and WKY LF saline compared to WKY HF saline at the end of the experiment (66 weeks of age).

	Palm^11^-PrRP31 vs. HF Saline		Liraglutide vs. HF Saline		LF Saline vs. HF Saline	
Metabolite	∆ [%]		∆ [%]		∆ [%]	
URINE						
1-Methylnicotinamide	−15.18		43.10	*	−28.33	
Sucrose	78.01	*	10.19		−60.34	
Creatinine	−18.73	*	−19.69	*	−27.38	**
Taurine	−30.07		−53.85	**	−78.03	*
β-alanine	−40.72	***	−44.33	***	−58.79	***
2-Hydroxyisobutyrate	−12.87		75.28	*	−2.03	
PLASMA						
Glucose	−30.89	**	−28.50	*	−32.94	**
Arabinose	−32.88	**	−31.52	*	−37.79	**
Sucrose	−22.62	^×^	−23.25	^×^	−33.34	**
Unidentified saccharide	−26.47	**	−27.30	**	−29.65	**
3-Hydroxybutyrate	−15.88		−49.10	*	−31.68	
Creatine	−31.10	**	−29.09	*	−33.61	**
Fumarate	−38.62	*	−29.62		−24.14	
Lactate	−45.52	^×^	−11.94		−40.01	
Glycine	41.66	**	7.44		23.63	
Serine	37.94	**	14.39		3.06	
Tryptophan	−21.25	*	−0.17		−5.27	

The results are presented as the percentage change of normalized concentrations in WKY HF palm^11^-PrRP31, WKY HF liraglutide and WKY LF saline vs. WKY HF saline groups (n = 6–8). The statistical significance was analyzed by one-way ANOVA. * *p* < 0.05, ** *p* < 0.01, *** *p* < 0.001, ^×^ trend with *p* < 0.1.

## Data Availability

The data presented in this study are available on request from the corresponding author.

## Supplementary Materials

The following are available online at https://www.mdpi.com/article/10.3390/nu15020280/s1, Figure S1: Scores plots of PCA and PLS-DA models of urine (at 60 weeks) and plasma (at 66 weeks) samples from WKY HF and LF rats; Figure S2: Volcano plot of liver lipids indicating lipid species that are significantly increased or decreased in the WKY rats on HF diet compared with WKY rats on LF diet. Figure S3: Volcano plot of liver lipids indicating lipid species that are significantly decreased in the WKY rats on HF diet treated with palm11-PrRP31 compared with WKY rats on HF diet treated with saline. Figure S4: Volcano plot of liver lipids indicating lipid species that are decreased in the WKY rats on HF diet treated with Liraglutide compared with WKY rats on HF diet treated with saline. Figure S5: Quantification of immunohistochemically stained slices for doublecortin, a marker of newly generated neurons in the hippocampal dentate gyrus. Figure S6: The concentration of blood plasma c-reactive protein (CRP) in fasting plasma (µg/mL). The data are presented as means ± S.E.M. Table S1: Significant changes of urinary and plasma metabolites in WKY HF compared to WKY LF rats.
