# Supplementary material for: Lipidized PrRP Analog Exhibits Strong Anti-Obesity and Antidiabetic Properties in Old WKY Rats with Obesity and Glucose Intolerance
Source: Nutrients. 2023 Jan 5;15(2):280. doi: 10.3390/nu15020280 (PMC9864151; doi:10.3390/nu15020280)
Supplement: Supplementary file 1 [file nutrients-15-00280-s001.zip › nutrients-2089635-supplementary.docx]

**Mrazikova et al**

**Supplementary Materials:**

**Supplementary Table 1.** Significant changes of urinary and plasma metabolites in WKY HF compared to WKY LF rats.

| ***Urine*** | **WKY HF vs. LF** | |  | ***Plasma*** | **WKY HF vs. LF** | |
| --- | --- | --- | --- | --- | --- | --- |
| **Metabolite** | **∆ [%]** | **p-value** |  | **Metabolite** | **∆ [%]** | **p-value** |
| 1-Methylnicotinamide | 43.08 | 1.32E-02 |  | Glucose | 49.12 | 2.75E-03 |
| Nicotinamide N-oxide | 114.29 | 1.03E-05 |  | Arabinose | 60.75 | 1.71E-03 |
| Nicotinamide | 133.01 | 5.25E-04 |  | Sucrose | 50.02 | 4.78E-03 |
| Nicotinurate | 29.72 | 4.62E-04 |  | Unidentified saccharide | 42.15 | 1.67E-03 |
| Trigonelline | -94.24 | 3.30E-07 |  | Leucine | -23.35 | 9.96E-03 |
| Putrescine | -39.72 | 5.24E-04 |  | Isoleucine | -29.55 | 1.28E-03 |
| Hippurate | -89.54 | 2.39E-06 |  | Valine | -15.21 | 5.43E-03 |
| 3-Indoxylsulfate | -48.78 | 1.30E-04 |  | Lysine | -15.07 | 1.47E-02 |
| Phenylacetylglycine | -51.46 | 1.03E-03 |  | 2-Hydroxyisobutyrate | 224.11 | 6.62E-06 |
| p-Cresylglucuronide | -42.13 | 3.75E-02 |  | 3-Methyl-2-oxovalerate | 75.77 | 2.73E-02 |
| Benzoate | -82.18 | 4.44E-04 |  | Creatine | 50.62 | 2.13E-03 |
| Glucose+derivatives | 49.86 | 8.78E-08 |  | Citrate | 19.31 | 1.71E-02 |
| Fucose | 41.98 | 4.54E-03 |  | Pyruvate | 63.42 | 3.38E-02 |
| Xylose | 51.50 | 1.73E-03 |  | Sarcosine | 22.22 | 5.51E-03 |
| Arabinose | 45.94 | 2.82E-05 |  |  |  |  |
| Sucrose | 220.28 | 3.87E-04 |  |  |  |  |
| Choline | 35.15 | 1.83E-03 |  |  |  |  |
| Methylamine | -46.94 | 4.88E-05 |  |  |  |  |
| Citrate | -33.84 | 1.29E-02 |  |  |  |  |
| Succinate | -22.32 | 3.19E-02 |  |  |  |  |
| 2-Oxoglutarate | -32.90 | 1.67E-02 |  |  |  |  |
| Fumarate | -48.67 | 1.59E-03 |  |  |  |  |
| Lactate + threonine | 32.64 | 1.35E-04 |  |  |  |  |
| Creatinine | 24.75 | 3.31E-06 |  |  |  |  |
| Taurine | 359.15 | 1.34E-06 |  |  |  |  |
| Isovalerylglycine | -45.96 | 1.78E-05 |  |  |  |  |
| Butyrylglycine | -32.64 | 3.41E-07 |  |  |  |  |
| Deoxyuridine | 79.40 | 3.97E-03 |  |  |  |  |
| Cytidine | 75.27 | 3.98E-05 |  |  |  |  |
| Pseudouridine | 9.81 | 6.52E-03 |  |  |  |  |
| N-acetyls of glycoproteins | 30.98 | 7.10E-05 |  |  |  |  |
| Beta alanine | 122.60 | 5.83E-05 |  |  |  |  |
| Tartrate | 2317.26 | 3.01E-05 |  |  |  |  |
| Lipids + bile acids | -45.88 | 1.03E-04 |  |  |  |  |

The results are presented as the percentage change of normalized concentrations in WKY HF vs. WKY LF groups (n=6-8). The statistical significance was analyzed by unpaired t-test.


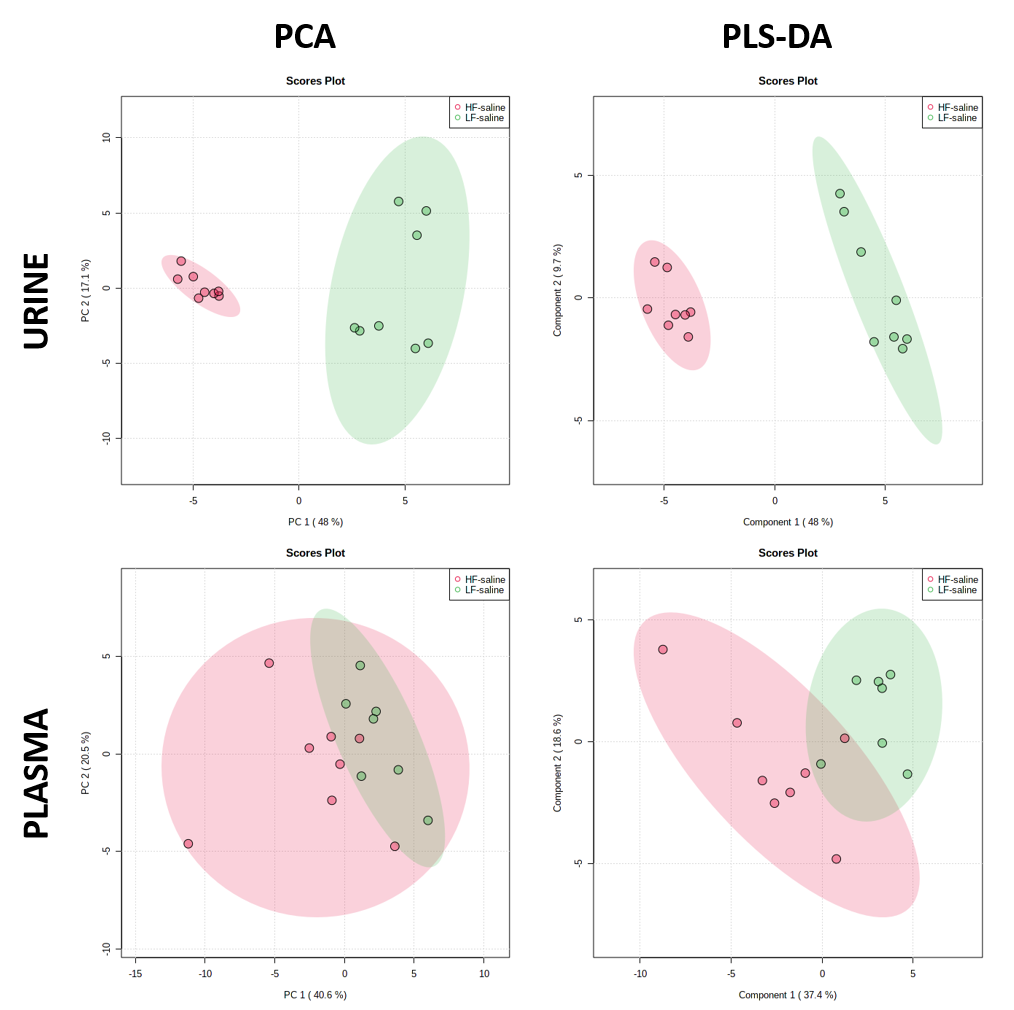


**Supplementary Figure 1.** Scores plots of PCA and PLS-DA models of urine (at 60 weeks) and plasma (at 66 weeks) samples from WKY HF and LF rats. The PLS-DA cross-validation results for 2 principal components: accuracy=1, R2=0.99, Q2=0.96 for urine; accuracy=0.80, R2=0.75, Q2=0.35 for plasma. WKY HF group is marked in red, WKY LF group in green.

**
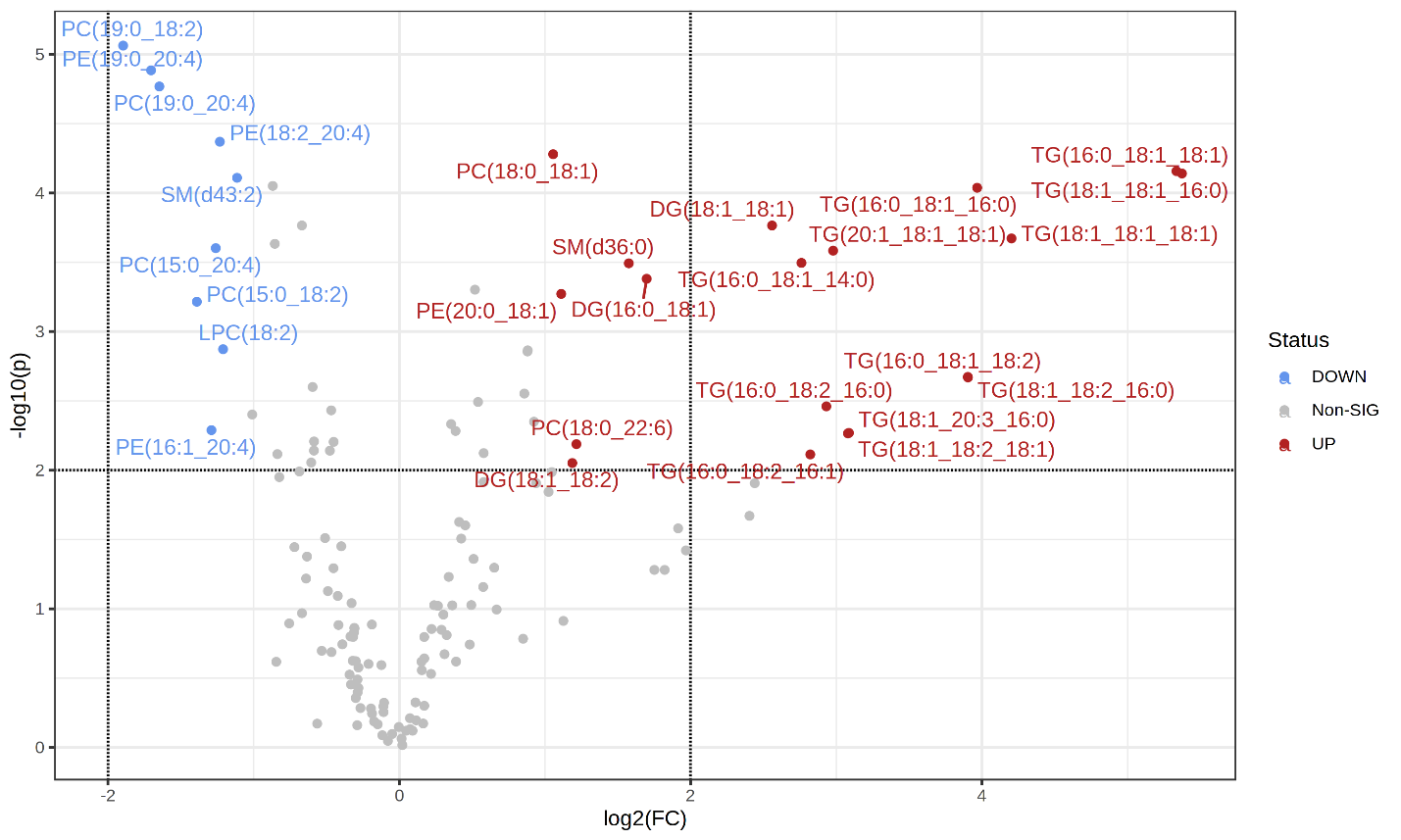
**

**Supplementary Figure 2.** Volcano plot of liver lipids indicating lipid species that are significantly increased or decreased in the WKY rats on HF diet compared with WKY rats on LF diet (fold change > 2 and p-value < 0.01 by Student’s t-test).

**
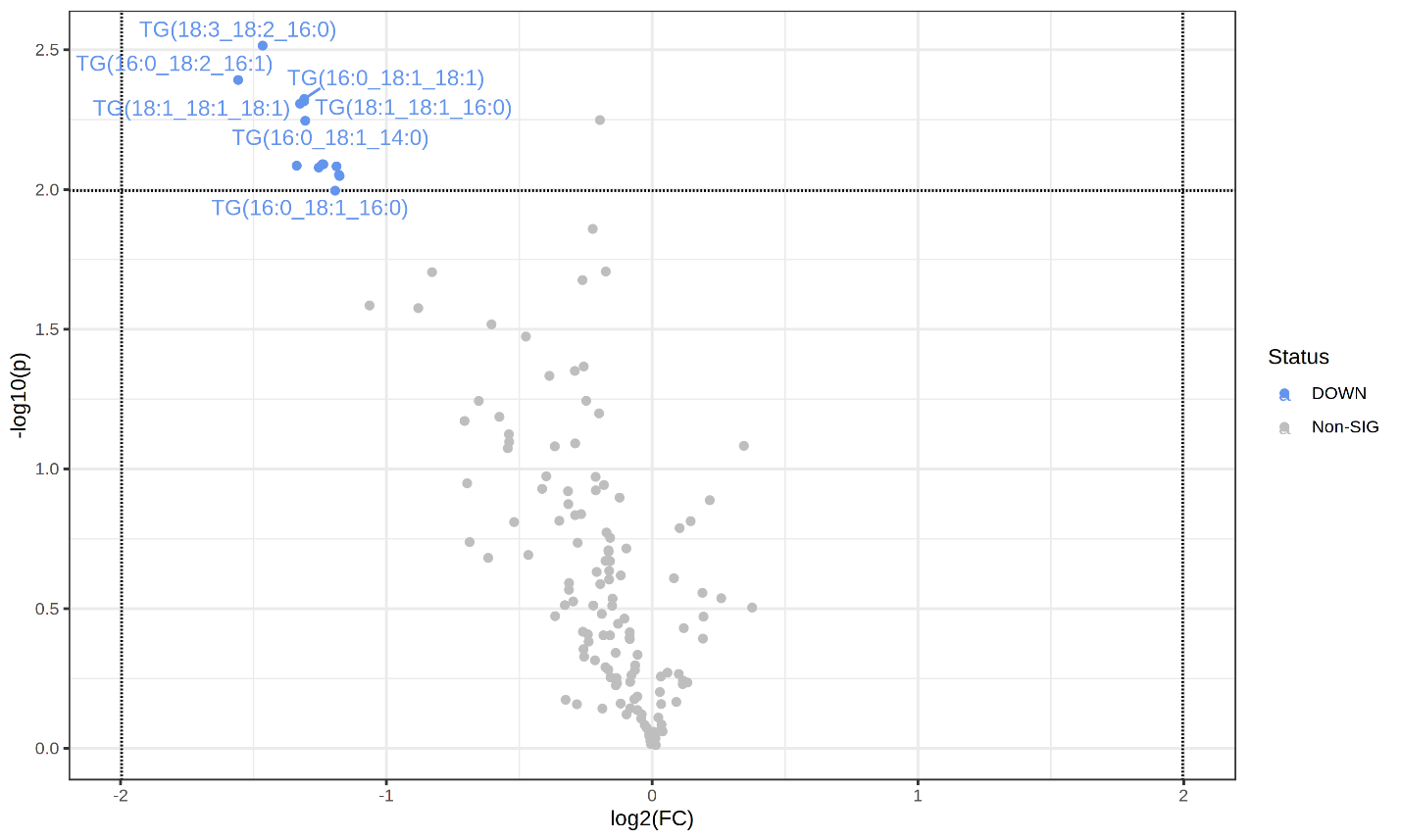
**

**Supplementary Figure 3.** Volcano plot of liver lipids indicating lipid species that are significantly decreased in the WKY rats on HF diet treated with palm^11^-PrRP31 compared with WKY rats on HF diet treated with saline (fold change > 2 and p-value < 0.01 by Student’s t-test).

**
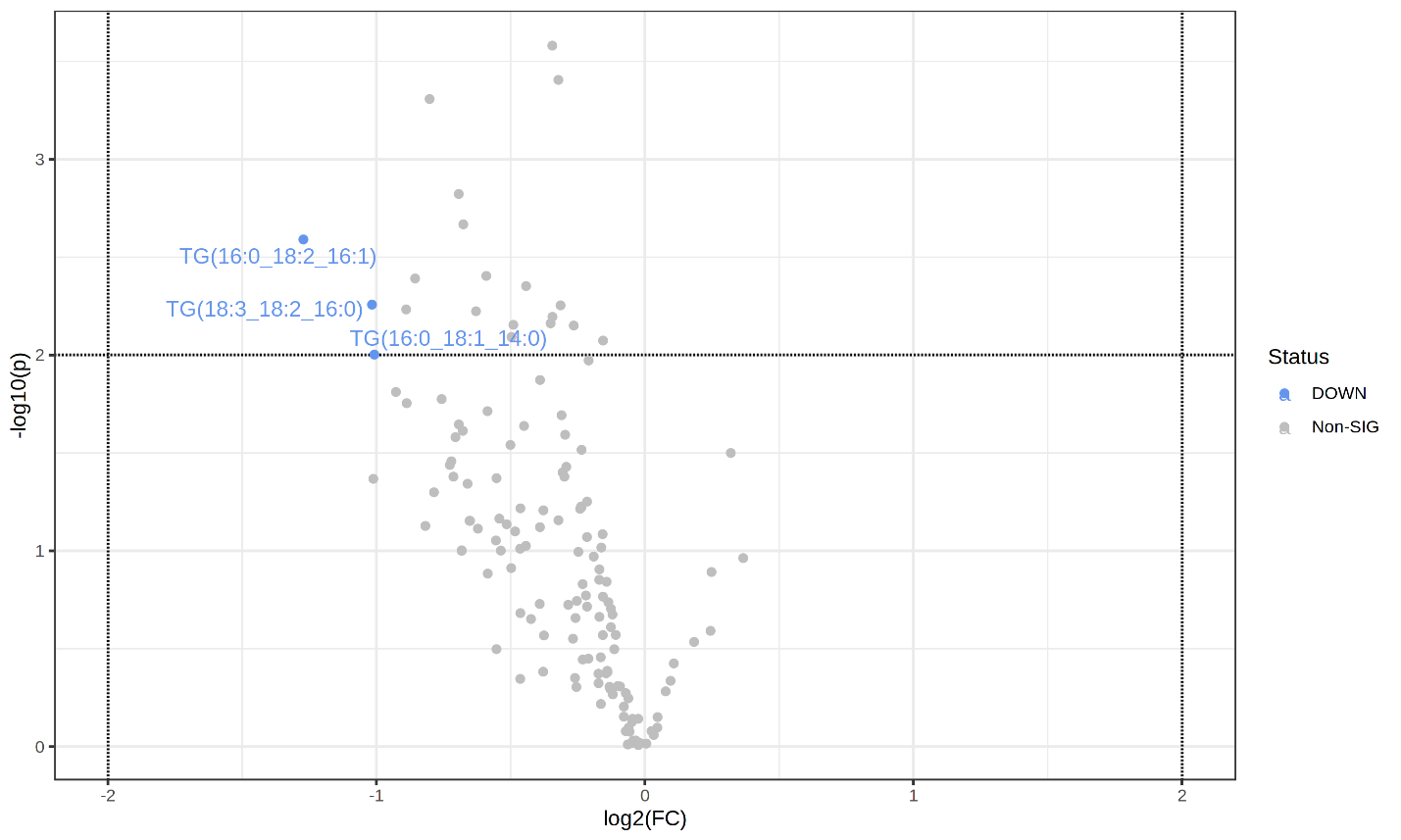
**

**Supplementary Figure 4.** Volcano plot of liver lipids indicating lipid species that are decreased in the WKY rats on HF diet treated with Liraglutide compared with WKY rats on HF diet treated with saline (fold change > 2 and p-value < 0.01 by Student’s t-test).


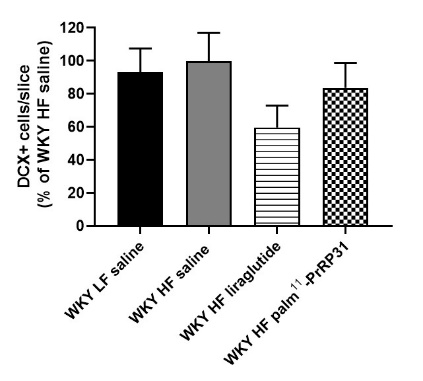


**Supplementary Figure 5.** Quantification of immunohistochemically stained slices for doublecortin, a marker of newly generated neurons in the hippocampal dentate gyrus. The data are presented as means ± S.E.M. Statistical analysis was performed by one-way ANOVA with Dunnett´s *post hoc* test (n= 5-6 rats per group).


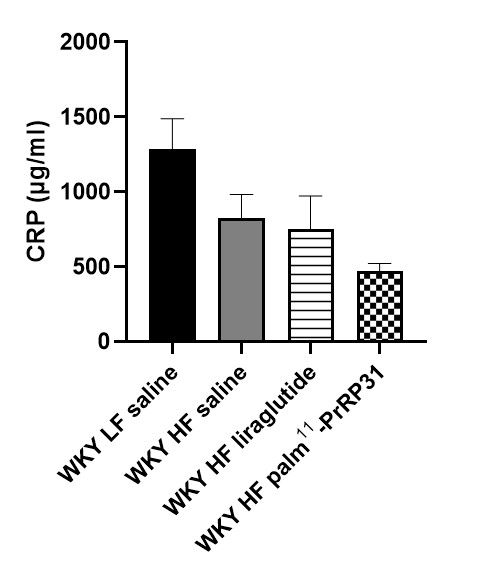


**Supplementary Figure 6.** The concentration of blood plasma c-reactive protein (CRP) in fasting plasma (µg/ml). The data are presented as means ± S.E.M. Statistical analysis was performed by one-way ANOVA with Dunnett´s *post hoc* test (n= 6-8).
